# Medical Miscellanies

**Published:** 1816-01

**Authors:** 


### PART IV.

### MEDICAL MISCELLANIES.

*It is a well known fact that those who are most extensively engaged in practice, and have the greatest opportunities of seeing and recording the phænomena of disease, are most averse to diffuse their knowledge through the press to their less experienced brethren. This is a great misfortune, and may be attributed to several causes, the principal one of which is, the dislike most men feel to lay themselves open to criticism or public scrutiny. Another is, the want of leisure to polish and arrange their observations, which they consider as indispensable before committing themselves to print. The* MISCELLANEOUS DEPARTMENT *of the Medico-Chirurgical Journal is meant to obviate these objections, by opening a medium for the communication of every useful fact or opinion in medicine, without subjection to censure or criticism, and without the trouble of artificial arrangement, or unnecessary ornament. The names of the contributors will repose in safety with the Editors, while the contributions shall be diffused in the simple language of truth, for the good of suffering humanity, and the advantage of the profession at large. The Editors invite their brethren to render this department of their Journal, though last in order, not least in utility or merit.*

### CHEMISTRY.

> " The urine, the saliva, or the bile, indifferently taken from such and such a subject, may be analysed, and hence results our Animal Chemistry; but the Physiology of our fluids should be composed of the innumerable variations which they experience, according to the different states of their respective organs."
>
> BICHAT.

THE excellent and ever to be lamented Author above quoted, has clearly shewn, that if any one member or part of the body be brought to more than ordinary perfection, some other part, or the whole, must suffer in proportion. He says the same of the faculties of the mind; and that to arrive at any thing like exclusive perfection in one science, must be at the loss of perfection in others. Such are the difficulties and delays in our education, the shortness of life, and innumerable other obstacles, that an eminent knowledge in the healing art cannot be acquired by one person in fifty, where great cultivation of an *auxiliary* science is aimed at. Of all the subordinate sciences, Chemistry has confessedly contributed the least to an accurate knowledge or discrimination of disease, and the means of alleviation. What remedy has all the brilliant modern discoveries in pneumatic Chemistry added to the list? Has the inhalation of oxygen or hydrogen gas, done any thing for the removal of human infirmities? Alas, No! Has Animal Chemistry done any thing in this way? If it be now the opinion that the blood is decarbonated, instead of oxygenated, in the lungs, can we cure pulmonary consumption the better? Without meaning to depreciate a delightful and entertaining science, may it not be safely asserted, that every hour employed in the *minutiæ of Chemistry* by the Medical Practitioner, produces a defalcation in the quantum of *practical knowledge*, which it is the duty at least of every man to endeavour to acquire, who professes the healing art.

MEDICUS.

---

### DR. SEEDS ON BLOODLETTING

We solicit the attention of the medical reader to the following Experiments, which, with others now making, of a similar nature, in different quarters, may lead to more important results than at first sight might be expected.

Concerning epileptic disorders, medical authors, from the commencement of our science to the present day, have offered many conjectures; but who is not aware that the real nature, and consequently the proper method of treating them, are equally involved in obscurity? As the difficulty and uncertainty of the subject are universally admitted, it is hoped that indulgence will be shown to any attempt, however humble, at throwing light on this interesting topic.

The experiments which are to be detailed, were carefully made, and every circumstance narrated as early as possible, so that their accuracy may be depended on, one or two professors always being present, and subsequently revising the details.

1st *Experiment*. The left crural artery of a healthy dog was divided. The struggles were incessant, and greatly accelerated the pulse, but he scarcely seemed to suffer pain, except on the first division of the integuments.

In little more than half an hour from the commencement, the artery of the right leg was divided; the vessel first opened at this time beat very languidly, and in a short time ceased to pour blood, whilst the other continued active to the last.

The heart beat very strongly, and its motions were distinctly perceptible over the whole thorax and abdomen. When the limbs had become cold, and no sign of life was present, the heart still palpitated, the respiration was still laborious. In an hour life ceased.

On dissecting the body, we found the spinal marrow pale, except at the top and bottom, where it was slightly red; on the contrary, the nerves on the points were whitest, and reddish in the middle. The sinus venosi were bloodless. The d. m. cerebri was slightly red, and one rather large blue vessel was seen on the corona.

The substance of the brain was very white, as were all the nerves, except the optics and first pair, whose base was slightly red.

The ventricles were so full of water, that till we had witnessed a similar result in other experiments, we were doubtful of its having been all effused during the experiment.

The right side of the heart was very soft, and contained some black clotted blood; the left was very hard and thick. The viscera were generally pale. Some of the mesenteric glands were full of a dark coloured liquid.

The palpitation of the heart, the most conspicuous symptom in this case, was very great; it plainly shows, in what state of the body such are likely to happen, and it likewise warns us not to judge of the vigour of the blood from a partial examination, for not unfrequently, when death is just at hand, the heart beats as tho' it would burst the thorax; at other times, when the smaller vessels are collapsed and deprived of vitality, the larger trunks are powerfully contracting.

Do we not see from this, that, for the due circulation of the blood the heart has need of a vigorous support from the arteries?

The weakness of the body was on a par with the difficulty of breathing, and both advanced with equal steps; hence it appears how intimately the functions of the lungs and blood-vessels are connected.

On dissecting the body, we found that every part was bloodless; especially the different parts of the nervous system.

Why, from opening those vessels, was the body so completely drained of blood? and particularly venous blood?

2d *Experiment*. We procured a small dog who had a wound on the right side, penetrating the lungs; from this wound he seemed to suffer little uneasiness during a week that he was in our possession.

The left crural artery was cut. The respiration was very much hurried, and the air was expelled with a loud hissing from the wound in the side. The strength of the heart's motion slowly and gradually diminished: once indeed, a sudden and rapid acceleration of the discharge of blood took place. The weakness of the pulse and dyspnœa were equal, and uniformly kept pace with each other. The legs were alternately bent and extended, and the tongue thrust out of the mouth and again drawn into it. The pupils became insensible to light, long before life ceased. In little more than half an hour, death put an end to his miseries.

On the following day, the body was examined. The d. m. of the brain was colourless and bright. A few small red vessels were seen on the surface of the brain, and amongst its convolutions; its substance and all the nerves were soft and white.

The plexus choroides was of a bright red; there was no blood in the head, except in the basilar veins, which contained a little.

The medulla spinalis could not be properly examined, owing to an unfortunate accident which occurred.

We could not inflate the right lung; the left presented nothing unusual. A few red spots were observed on the stomach. There was a little red blood in the mesentery and omentum. The colon was somewhat contracted, the small intestines crepitated on being handled. The urinary bladder was hard, contracted, and empty.

No one doubts that the integrity of the functions of the lungs is necessary to the health of the body; it appears equally true, that the more readily the blood is received into the lungs and returns through them to the heart, the more completely do we enjoy health. This fact is of the utmost importance both to the student and practitioner of medicine.

This animal laboured under a wound of the lungs, which impeded their functions and interrupted the ordinary changes of blood; thus, though he lost less blood, he was sooner weakened, and much sooner destroyed, than the subject in the preceding experiment.

The irregularities of muscular action were more evident, both in the voluntary and likewise in the involuntary muscles.

Does not this experiment teach us, that in some cases of diseases of the lungs, an excessive venæsection may prove injurious?

3d *Experiment.* An incision was made into the subclavian artery of a small dog. The pulsation of the arteries were immediately quickened, and rose at first to 130, and not long after to 156 in the minute.

The motions of the heart were languid, but regular; they had ceased long before death. In less than a quarter of an hour, the pupils were dilated and motionless.

The breathing soon became slow and heavy, and at the same time the neck was slightly twisted. In twenty minutes, death supervened.

The thorax was first examined. The right side of the heart was empty, and flaccid; almost all the large veins were full of blood; and in the left side of the heart, and in the abdominal aorta, there was a little dark blood.

Most of the abdominal viscera were pale. In the spleen and pancreas were many dark spots. A very small portion of one of the ureters was ossified. The nerves every where were white and shining. The d. m. c. was colourless. Almost all the sinuses were dyed with blood. Many red and blue vessels were seen among the convolutions of the brain. The plexus choroides was of a bright red. The whole surface of the brain was moist. There was some degree of redness in the spinal marrow, especially in the lower part, as well as of all the nerves. The external coverings of the cord were nearly colourless. No dark coloured blood was to be seen.

In this instance the prostration of the strength was greater and more rapid, and life was sooner extinguished than in either of the former examples.

The subclavian artery giving off the vertebral, it was to be supposed would, if wounded, speedily affect the head.

Why where the pulsations of the heart so soon, and so greatly accelerated? Why so much more venous blood left in the body than before?

(To be continued.)

*Extract of a Letter from Mr. Golding to the Editors.*

"Among the many valuable additions made in these latter ages to the curative means employed by the physician, Electricity claims a prominent rank; yet it must be confessed, that the extent of its remedial powers has never hitherto been duly appreciated, nor a knowledge of its specific effects upon the animal economy so extensively disseminated as the subject deserves. This is in some measure to be accounted for from the administration of the electric fluid, as a medical agent, having been for the most part confined to unskilful and unprofessional hands; whilst those few persons who have employed it with science and judgment, have either been too much occupied with pursuits they deemed more important, or used this power on too limited a scale, to allow them to give a comprehensive view of its effects and mode of action. To supply this deficiency, I propose, if it should meet with your approbation, to communicate from time to time, through the medium of your Journal, the results of such interesting cases, whether successful or otherwise, as shall come under my management during the time I conduct that department of practice in the Westminster General Dispensary; in which Institution, Electricity is very extensively prescribed, in all its various forms, as a means of cure for numerous diseases. The experience I have already had of this remedy, during the years 1813 and 1814, whilst its administration was entrusted to my management in St. Thomas's Hospital, has impressed me with a favourable opinion of its efficacy; and the extensive opportunities now afforded me, will enable me to subject that opinion to the test of rigorous examination. The mass of facts thus brought forward, will, it is presumed, be serviceable to my professional brethren, by pointing out, on the basis of experimental observation, those maladies to which Electricity is peculiarly adapted, as also those in which it is found to be manifestly hurtful and injurious." —— Electricity, as a branch of medical practice, is not, we are of opinion, by any means yet understood by practitioners in general: nor has it obtained that share of attention to which it is justly entitled. We shall gladly avail ourselves of the opportunity of bringing its merits fairly before the Public, and shall therefore, with pleasure, insert such interesting and important cases as we may be favoured with by the writer of the above letter. Edit.

*Extraordinary Operation.* Wepser relates the case of a peasant, who, long affected with severe head-ach, consequent, as the result proves, on an effusion of lymph between the cranial bones and dura mater, applied to a blacksmith. This man, who was in the habit of trepanning cattle for the Gid, consented, at the urgent solicitation of the peasant, to perform a similar operation upon him. An opening was made in the sinciput by means of a carpenter's whimble, without any of the precautions observed by regular operators. A quantity of serous fluid issued from the orifice, and the peasant was completely cured. *Dictionaire des Sciences Medicales.*

In the memorable battle of Leipsic, a soldier received a musket-shot in the head, near the union of the parietal with the squamous portion of the temporal bone. The foreign body had evidently penetrated within the cranium, where it remained lodged. The soldier complained only of constant and severe head-ach; and occasionally experienced epileptic paroxysms. Eleven months after the infliction of the wound he died; and on examination of the encephalon, the bullet was found on the tentorium cerebelli, completely imbedded in the substance of the brain. *Hufeland's Journal der Practischen Heilkunde.*

On the 30th of November, St. Andrew's day, the Royal Society held their annual meeting, at their apartments in Somerset Place, when the President, the Right Hon. Sir Joseph Banks, in the name of the Society, presented the gold medal (called Sir Godfrey Copley's) to David Brewster, LL. D. for a paper "On the Polarisation of Light by Reflection from transparent Bodies," printed in the last volume of the Philosophical Transactions. After which the Society proceeded to the choice of a Council and Officers for the ensuing year, when, on examining the lists, it appeared that the following gentlemen were elected:—*Of the old Council.* The Right Hon. Sir Joseph Banks, Bart. K. G. C. B. Sir Charles Blagden, Knight, Samuel Goodenough, Lord Bishop of Carlisle, Taylor Combe, Esq. Davies Giddy, Esq. M. P. Sir Everard Home, Bart. Samuel Lysons, Esq. George Earl of Morton, K. T. John Pond, Esq. William Hyde Wollaston, M. D. Thomas Young, M. D.—*Of the new Council.* John Barrow, Esq. Mark Beaufoy, Esq. Henry Browne, Esq. Sir Humphrey Davy, Knt. Philip Earl of Hardwicke, K. G. Edward Howard, Esq. John Latham, M. D. Pres. Coll Phys. Thomas James Mathias, Esq. Sir John Nichol, Knt. M. P. George Earl of Winchelsea, K. G.—*Officers.* President, the Right Hon. Sir Joseph Banks, Bart. K. G. C B.—Treasurer, Samuel Lysons, Esq.—Secretaries, William Hyde Wollaston, M. D. and Taylor Combe, Esq.

At one of the late meetings of the Linnæan Society, an account was given of a considerable number of specimens of cinchona which had been taken in a Spanish Ship. Five of these were not referable to any known species, but appeared to be new. The yellow bark of the shops is to be obtained from the *cinchona hirsuta* of the *flora Peruviana.*

On Wednesday the 6th of October, an election took place at the City Dispensary for a Physician to that Institution in the room of Dr. Sayer Walker, who has resigned. Three gentlemen originally started for the vacant office, but two only stood the poll, Dr. Uwins and Dr. Martin; upon casting up the votes a majority was found to be in favour of Dr. Uwins, who was accordingly declared duly elected. It is, we understand, a regulation of this Establishment, that the successful candidate shall within a year after his election procure 400 new subscribers to it, or, in other words, pay down four hundred guineas. How far the adoption of such a regulation is liberal on the part of a public charity, or a compliance with it, consistent with the proper dignity of the profession, it is not for us to determine. The salary of this office is, we believe, one hundred pounds *per annum.*

A Gentleman, whose constitution of mind must be more than ordinarily sanguine, has proposed a plan, as novel in its conception, as impracticable in its execution, for the entire extirpation of the Venereal Disease; an object which he thinks might, with the countenance and support of the legislature, be accomplished in the course of a few months. After stating the serious inconveniences and enormous expences sustained by the military and naval departments of the public service by the extensive ravages of this disease, the projector thus developes his plan of eradication.

"For this purpose, I would propose, that in London, Dublin, Edinburgh, Portsmouth, Plymouth, Chatham, Cork, Liverpool, Bristol, &c. where it chiefly exists, and whence it is disseminated, a temporary Asylum, (some unoccupied rooms in any public building, where, in the summer season, no furniture would be necessary, but what they themselves could bring) should be allowed to Girls of the Town, where, attended by Medical Men employed by Government, (or the different counties as may be) they could be secluded until perfectly well; this, at the same time, would be a very proper period to instil principles of reformation among them. They would gladly embrace the opportunity of getting rid of their greatest annoyance; and if even one or two, from perverseness, or some foolish whim, should hold back, as no secrets are kept among the frail sisterhood, they would instantly be pointed out by the others, when the least hint from a Constable would be sufficient to enforce obedience. To act in unison with this part of the plan, certain questions ought to be put to every master of a vessel as to the state of his crew, in this way, on his arrival in any port; for although this disease, I apprehend, is seldom imported, but much oftener exported, (for many reasons) yet it would be well to guard every avenue to its access. All other men, in civil life, who wantonly extend this plague, should be made liable to some fine or mark of infamy; and it would be no difficult matter, from concomitant circumstances, to prove the fact. They might be placed, as dangerous men, *under the superintendance of the Police.* With our present knowledge of the advantages of vaccination, it is justly considered highly penal to innoculate for the small-pox;* it ought not to be held less so, the wilful propagation of any other pestilence. The very liability to be brought forward on such a charge, would deter almost every man from the commission of this crime. It fortunately happens, that the grand source from whence this virus

* This is a mistake; there is no penalty for inoculating with the small-pox, but there is for incautiously or wilfully exposing an inoculated patient so as to hazard the communication of the disease to other persons.
EDITORS.

is kept up and renewed, can be most easily watched, and, *to a certainty*, cut off. The seamen and soldiers in sea-port and garrison towns, who keep this disease in constant circulation, may, without any difficulty, be prevented from spreading it, by the surgeon of every ship or regiment answering for the men under his immediate charge, by adopting such regulations that no man can conceal his situation, and by carefully securing, during their cure, all those found infected.

"The medical men undertaking the management of this affair would, on many accounts, be best selected from the half-pay of the army and navy—one or two only would be necessary for any of the towns mentioned, except the capitals. Let their salary, (added to their half-pay) be in the first instance moderate; but let credit and reward *depend upon the success of their exertions;* and if they succeeded in banishing from the land one of its greatest pests, they would well deserve both honour and emolument. They should be armed with certain powers of scrutiny and investigation among the proper objects, to be used with all possible delicacy, and even with kindness.

"The management of the great towns of the interior might be committed to their respective magistrates and guardians of the poor, for they feel (in poor-rates, &c.) as well as the army and navy, the effect of premature decrepitude, brought on by this disorder. It also insinuates itself by circuitous routes into the most virtuous families in the kingdom, and embitters their peace—all, therefore, are equally interested in promoting its extinction. A very short time only would be necessary for this purpose. By vigorous and simultaneous efforts on the parts of the magistracy, the heads of departments in the army and navy, it is extremely probable that syphilis would be unknown at the end of the summer, throughout the whole of both islands. But the co-operation must be cordial, commencing together, at the beginning of the warm weather, and thus the enemy would be rooted out in every quarter at the same time, and prevented from nestling any where."

The author seems to be aware, that his scheme may meet with opposition, and among its opponents he does not fail to include *some* medical men, who at present derive considerable emolument from their practice in this disease and its consecutive affections.

A Society is just formed in the Metropolis, under the name of "The Medical Annuitant and Benevolent Society," for the purpose of raising, either by single payments or annual subscriptions, an adequate fund to allow an annuity of not less than £.50 to such of its members as shall have attained the age of 60 years, and for relieving those under that age who shall have unavoidably fallen into distressed circumstances. Any physician, surgeon, or apothecary, residing and regularly practising his profession within England and Wales, is eligible to be admitted into this Society.

Dr. Halliday, of Birmingham, has issued proposals for publishing, in one vol. 8vo. a Translation of "The Doctrine of Incitement, illustrated by Dr. Frank, Professor of Medicine in the University of Vilna, Poland, &c."

The Spring Courses of Lectures at St. Bartholomew's Hospital will commence on Saturday, Jan. 20, 1816. On the Theory and Practice of Medicine, by Dr. Hue. On Anatomy and Physiology, by Mr. Abernethy. On the Theory and Practice of Surgery, by Mr. Abernethy. On Chemistry and Materia Medica, by Dr. Hue. On Midwifery, by Dr. Gooch. Anatomical Demonstrations, by Mr. Stanley. Further particulars may be obtained by application to Mr. Wheeler, apothecary to the Hospital.

Dr. Clutterbuck will begin his Spring Course of Lectures on the Theory and Practice of Physic, Materia Medica, and Chemistry, about the middle of January, at ten o'clock in the morning, at his house, No. 1, in the Crescent. New Bridge Street, where further particulars may be had.

Mr. Taunton's Course of Lectures on Anatomy, Physiology, Pathology, and Surgery, will commence on Saturday, Jan. 27, 1816, at eight o'clock in the evening precisely, and be continued every Tuesday, Thursday, and Saturday, at the same hour. Particulars may be had on applying to Mr. T. 87, Hatton Garden.

Died. — At Bexley, in Kent, Thomas Latham, M. D. — At Watford, Herts, aged 63, Dr. Charles Kilby; he is succeeded in his practice by Dr. Andrews, late of the island of Madeira.

